# On the Complexity of Aggressive Behavior: Contextual and Individual Factors in the Taylor Aggression Paradigm

**DOI:** 10.3389/fpsyt.2019.00521

**Published:** 2019-07-25

**Authors:** Carmen Weidler, Ute Habel, Philippa Hüpen, Dilsa Akkoc, Frank Schneider, Julie A. Blendy, Lisa Wagels

**Affiliations:** ^1^Department of Psychiatry, Psychotherapy and Psychosomatics, Faculty of Medicine, RWTH Aachen University, Aachen, Germany; ^2^JARA-Institute Brain Structure Function Relationship (INM 10), Research Center Jülich, Jülich, Germany; ^3^University Hospital Düsseldorf, Düsseldorf, Germany; ^4^Department of Systems Pharmacology and Translational Therapeutics, Perelman School of Medicine, University of Pennsylvania, PA, United States

**Keywords:** aggression, Taylor Aggression Paradigm, aggressive behavior, sex differences, provocation

## Abstract

As many paths lead to aggression, understanding which situations and which person-specific traits facilitate or impede aggressive behavior is crucial. Provocation is among one of the most frequently reported predictors of aggressive behavior. However, it remains unclear whether the reaction to provocation is universal across different forms of aggression and whether individuals differ in their reactivity to such signals. Using the Taylor Aggression Paradigm (TAP), we investigated the influence of individual and contextual factors on physical and non-physical aggression in healthy men and women. The impact of trait aggression, sex, provocation, and the success of a competition against a fictitious opponent on aggressive behavior was examined in three different versions of the TAP. While equal provocation and punishment modalities were used in the first two versions, monetary deductions in the first and heat stimulus in the second study, the third experiment used non-physical provocation to trigger physical punishment. Trial-by-trial analyses revealed that provocation, independent of its specific nature, is a strong predictor for aggressive behavior, especially in highly aggressive participants. Although women initially showed less aggression than men, sex differences were diminished under prolonged, increasing provocation when provocation and punishment modality were identical. Only when modalities diverged, women, compared with men, were more hesitant to punish their opponent. These results, thus, extend evidence that women show lower levels of aggression under low provocation. However, high levels of provocation have similar effects on males’ and females’ reactive aggressive behavior across different forms of aggression. When competing for money, losing against the fictitious opponent was functioning as an additional provocative signal stimulating aggressive responses. Differences in aggressive responding have to be interpreted in the context of the specific type of provocation and aggression that is investigated since these modalities are shown to interact with individual characteristics.

## Introduction

Aggression is a biologically deep-rooted pattern of behavior and primarily serves to acquire and protect resources. However, manifested abnormally, human aggression can be problematic, prompting many researchers to investigate the underlying mechanisms. For a better understanding of these mechanisms, it is crucial to know what characterizes an individual who is likely to act aggressively, and, in addition, circumstances which contribute to the occurrence of aggression.

As a complex, multifaceted construct, aggression has been subject to many theories and definitions over the past years. Among the most popular definitions is that of Baron, who defined human aggression as “any behavior directed toward the goal of harming or injuring another living being that is motivated to avoid such treatment” (1).

Early aggression models, such as the frustration-aggression hypothesis (FAH), introduced by Dollard and colleagues in 1939, claim that aggression is always preceded by frustration (2). Later, this was modified into frustration being a potential trigger for aggression (3). Nevertheless, early models have often been criticized for omitting other factors apart from the frustrating event as elicitors of aggressive behavior. Subsequently, a number of domain-specific theories and overlapping models of aggression have been developed to explain etiology of aggression and psychological processes related to this behavior [e.g., Ref. (4)].

A contemporary and most commonly applied model, the General Aggression Model (GAM) (5), incorporates various factors that potentially influence aggressive behavior and provides a theoretical framework across different domains of aggression. Among biological and environmental factors, the model elucidates situational and personality factors that contribute to aggressive responding. The GAM further awards importance to individuals’ present internal state, comprising cognition, affect, and arousal, which potentially impacts appraisal and decision processes. The framework suggests that contributing factors are likely to interact.

In laboratory settings, factors contributing to aggressive responding have been investigated using different paradigms. Within such experimentally controlled scenarios, participants usually display different levels of measurable aggressive behavior. Most paradigms, for instance, the Point Subtraction Aggression Paradigm (PSAP) (6), the Taylor Aggression Paradigm (TAP; also known as the Competitive Reaction Time Task) (7), or the Ultimatum Game (UG) (8), involve social provocation to trigger aggressive responses in participants. Although the UG is usually applied to investigate the concept of fairness and self-interest, and thus may not directly measure aggression, the PSAP and the TAP are known as typical provocation paradigms that measure predominantly reactive aggression. Reactive aggression refers to impulsive and hostile forms of aggression, usually following provocation, as opposed to proactive aggression, which refers to instrumental and goal-directed behavior (9).

In the original version of the TAP, participants compete against an ostensible opponent in repeated reaction time tasks (7). The reaction time task is usually implemented to provide a competitive context that enables the investigation of potential aggressive behavior in a social situation. At the beginning of each trial, participants are instructed to select an electric shock intensity to be administered to their opponent in case the subsequent reaction time task is won. Likewise, participants themselves receive an electric shock after losing a trial with an intensity chosen by the apparent opponent. The selected intensity serves as the provoking stimulus and—selected by the participant—as a measure of aggression. Using the TAP, studies have consistently shown that provocation is closely linked to aggressive reactions. Following diverse provocation modalities, including monetary reductions (10–13), unpleasant thermal stimuli (14), pneumatic pressure (15), and aversive noise blasts [e.g., Refs. (16–18)], individuals select higher punishment levels for their fictitious opponent. Overall, research provides indications for a universal, modality-independent character of provocation as a predictor for aggression. However, it remains unclear whether some individuals, for instance, with high trait aggression, react differently to physical and non-physical provocation and whether other contextual factors, such as the outcome of the reaction time competition, affect aggressive behavior and the reactivity to provocation in different settings.

There is further an existing body of research reporting higher physical aggression in males than females. Meta-analyses showed higher levels of aggression under unprovoked conditions (19) and higher levels in physical aggression in real-world settings in men compared with women (20). Important findings in the meta-analysis were reduced sex differences under provoked conditions and larger sex differences regarding physical as compared with verbal aggression (19). Studies that investigated different forms of aggression, have shown similar levels of indirect aggression for men and women (21, 22). These mixed findings suggest that sex differences might differ for distinct forms of aggression and/or different situational properties. It is still unclear if males and females diverge in their reactivity, for instance, to lost competitions or different provocation modalities.

High trait aggression may predict the likelihood to engage in aggressive interactions throughout different contexts [e.g., Refs. (23–25)]. Within various aggression paradigms, the administered punishment level correlates positively with anger and aggressive traits (26–28). Yet, a meta-analysis emphasized that although personality traits are important modulators, their influence differs depending on the context. Specifically, while aggressiveness and irritability traits influence aggressive behavior under provoked and neutral conditions, trait anger, for instance, impacts aggression only under provocation (29). Although the association between aggressive traits and provocation has been investigated in several studies, modality-specific associations are still rarely studied. Further, it is not yet apparent whether aggressive traits also impact aggressive behavior through altered reactivity to other contextual factors. Specifically, highly aggressive individuals might react stronger to lost competitions, which could potentially present an additional provoking signal.

Provocation is probably one of the most powerful context factors. It modulates not only behavior but also affective states of individuals. Chermack and colleagues compared aggressive responses and positive and negative emotions during either low, constant, or increasing provocation by a fictitious opponent. Aggressive behavior and emotions associated with harm were higher in the increasing provocation condition (30). However, many studies do not investigate whether engaging in a social provocation paradigm actually impacts individuals’ affective state and what exactly leads to affective changes.

With the current study, we aim to investigate contextual and personality influences on aggressive behavior displayed in a laboratory setting. In three different versions of the TAP, we discretely examine the influence of contextual (physical and non-physical provocation, the outcome of the repeated reaction time competition against the fictitious opponent) and individual (trait aggression, sex) factors and their interaction on aggressive responses on a trial-by-trial basis. Further, emotional reactivity to different provocation modalities is investigated assuming an increase in negative emotions and a reduction in positive emotions following the provocation task.

**Figure 1:**
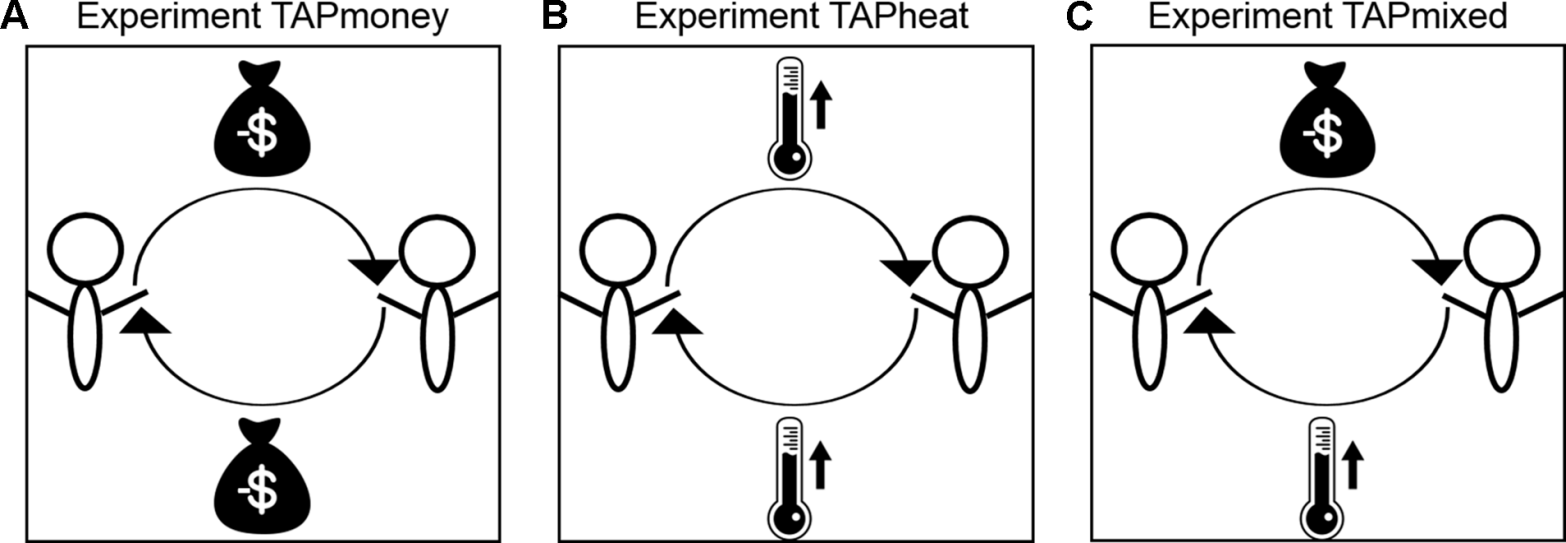
The figure depicts the three different versions of the TAP. **(A)** In experiment TAPmoney, participants are provoked by monetary deductions and are able to punish their opponent by money withdrawal. **(B)** In the TAPheat experiment, heat stimuli are used as the provocation and punishment stimuli. **(C)** In the TAPmixed experiment, participants are provoked by money subtractions and are able to punish the opponent with heat stimuli.

In the first study, participants are provoked by monetary subtractions from their account and are able to punish the fictitious opponent also using monetary deductions. In the second study, heat stimuli are used to provoke participants, who are able to retaliate selecting different intensities of heat stimuli. In the third study, participants are provoked by monetary subtractions but are able to punish their opponent with heat stimuli. A depiction of the three experiments is shown in **Figure 1**.

Assuming that provocation is a universal trigger, we expect higher punishment selections following high provocation in males and females. We further predict women to act less aggressively than men with sex differences being larger in experiments involving physical aggression. Additionally, we assume individuals with high trait aggression to react stronger to provocation as compared with low aggressive individuals.

## Methods

### Participants

In total, 81 healthy, right-handed participants were recruited using public advertising. All subjects gave their written informed consent in accordance with the Declaration of Helsinki prior to the experiment and were paid for participation. Twenty-seven volunteers participated in experiment 1 (13 female, mean age = 28.81 years; *SD* = 6.102), 27 in the second experiment (13 female, mean age = 25.33 years, *SD* = 2.449), and 27 in the third experiment (14 female, mean age = 24.07 years, *SD* = 4.753). The sample size for each group was determined according to previous studies using a similar modified version of the TAP (11, 31). The study was approved by the internal review board of the medical faculty of the RWTH Aachen.

### Questionnaires

Participants completed the Buss Perry Aggression Questionnaire (32) (AQ) and the Positive and Negative Affect Schedule (33) (PANAS). A self-developed questionnaire assessed whether participants followed a strategy. Using an open question strategy, was not defined in more details; therefore, participants could describe any kind of tactic. Due to the heterogeneity of responses, answers were categorized as either yes—the participant followed any strategy or no—the participant did not follow any strategy. The questionnaire further assessed whether they had any suspicions about the opponents’ participation in the study. AQ total and subscale scores for all participants of the three experiments are presented in **Table 1**.

**Table 1:** Trait aggression (Mean ± SEM).

		Males	Females	*p*
Money	N	14	13	
	Age	27.93 ± 1.13	29.77 ± 2.14	.456
	AQ total	64.54 ± 3.25	56.15 ± 2.96	.069
	Anger	15.23 ± 1.26	15.23 ± 1.26	1
	Hostility	16.62 ± 0.98	15.08 ± 1.43	.383
	Verbal	14.31 ± 0.79	14.31 ± 0.80	1
	Physical	18.38 ± 1.08	11.54 ± 0.80	<.001
Heat	N	14	13	
	Age	25.00 ± 0.75	25.69 ± 0.57	.474
	AQ total	66.45 ± 5.13	53.17 ± 4.50	.064
	Anger	16.18 ± 1.74	13.86 ± 1.67	.341
	Hostility	17.00 ± 1.38	15.25 ± 1.4	.288
	Verbal	14.14 ± 0.94	12.67 ± 1.10	.335
	Physical	18.62 ± 1.47	11.42 ± 0.77	.001
Mixed	N	13	14	
	Age	23.46 ± 1.16	24.64 ± 0.87	.419
	AQ total	62.85 ± 4.51	58.36 ± 4.05	.465
	Anger	14.69 ± 1.46	14.29 ± 1.26	.834
	Hostility	17.08 ± 1.60	17.79 ± 1.86	.776
	Verbal	12.54 ± 0.92	12.71 ± 0.92	.894
	Physical	18.54 ± 1.47	13.57 ± 0.78	.005

AQ, Aggression Questionnaire; SEM, standard error of mean.

### Procedure

In all experiments, participants were informed that the study aimed to investigate the relationship of attention and emotion processing to prevent a priming effect on the behavior displayed by participants in the subsequent task. Prior to the experiment, participants completed the AQ and the PANAS. Subsequently, participants were introduced to their same-sex opponent, a confederate of the experimenter, and they jointly listened to the instructions outlining the following reaction time tasks.

In experiments 2 and 3, the tolerance and pain thresholds of all participants were determined before participants were seated in front of the computer to perform the task.

Following the experiment, participants completed the second PANAS and their beliefs about the study were assessed. Thereafter, the experimenter verbally checked the credibility of the cover story. After completing the whole study procedure, all participants were fully debriefed about the nature of the experiment.

### Taylor Aggression Paradigm

At the beginning of each trial, participants could choose a punishment level. The following screen presented the opponent’s punishment selection, followed by an exclamation mark, signaling the upcoming reaction time task. Upon appearance of a visual cue, the participant should respond as fast as possible. Subsequently, participants were informed whether they lost or won the reaction time task. Simultaneously, participants heard a brief auditory feedback. In case of a lost reaction time task, the participant received the punishment selected by the opponent. The experiment predefined a winning rate of 50%. However, if participants responded faster than 200 ms or slower than 350 ms, the preprogrammed setting reset and participants won or lost the trial, respectively. The TAP consisted of 90 trials, equally distributed across three runs. Provocation increased from run 1 (range, 0–40, *M* = 20) to run 2 (range, 30–70, *M* = 50) to run 3 (range, 60–100, *M* = 82). Each run also included a few outliers (e.g., high provocation in run 1, low provocation in run 3).

**Figure 2:**
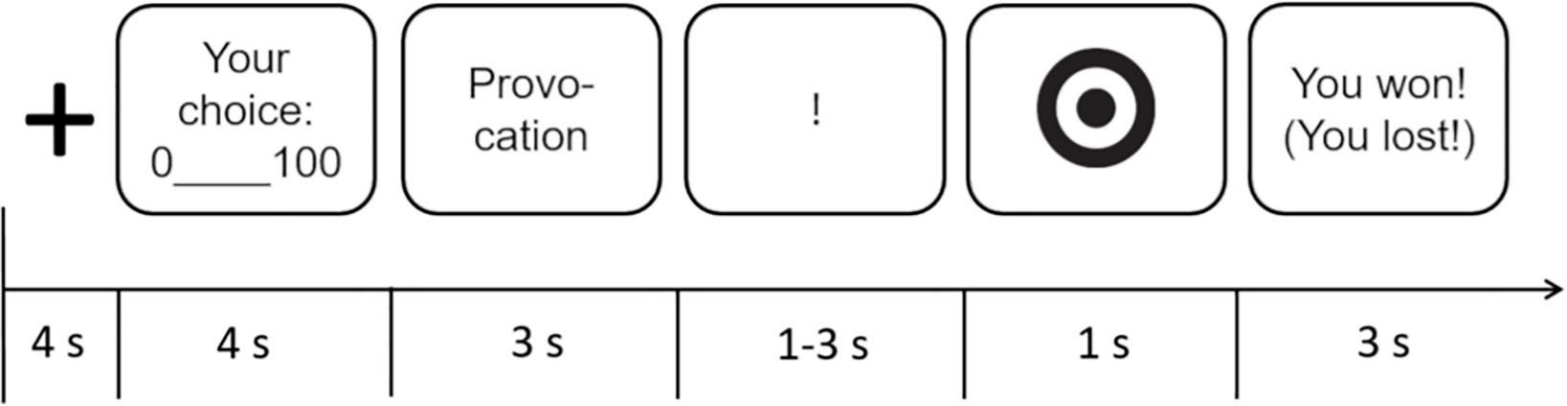
The illustration presents the sequence of a single trial of the TAP. Each trial started with a fixation cross, followed by the participants’ punishment selection. Subsequently, they were informed about the punishment selection of their apparent opponent. The exclamation mark informed participants to prepare for the reaction time game. After the response to the target, the outcome of the game was displayed (won/lost).

The trial sequence was constant across experiments and is shown in **Figure 2**.

In the first experiment, monetary subtractions were applied to provoke aggressive behavior (TAPmoney). At the beginning of each trial, participants could choose a punishment level ranging from 0 to 100 cents in steps of 10. Individuals started with 20€ in stock and were informed that by winning a trial, they would earn a randomized amount of money between 0 and 1€. In lost trials, the participant lost the monetary amount selected by the opponent. All participants finished the experiment with 2.30 €.

In experiment 2, slightly painful heat stimuli served as the provocation stimulus from the ostensible opponent and as punishment modality for the participant (TAPheat). Stimuli with intensities from 1 to 10 were administered to the inside of the left forearm approximately 1 cm below the elbow. The assessment of the tolerance and pain thresholds, as well as the delivery of the heat stimuli, was performed using the pain and sensory evaluation system “Pathway” from Medoc Ltd (Medoc, Ltd., Ramat Yishai, Israel). Using a contact heat-evoked potential stimulator (CHEPS), a small-area stimulus device with a circular contact area of 27 mm in diameter, heat stimuli were applied to the skin. The baseline temperature of the stimulator was set to 32°C. The highest punishment level equaled the previously acquired tolerance level of each individual and level 1 equaled that temperature minus 9°C, so that with each punishment level, the temperature was increased by 1°C. The applied stimuli ranged from 32°C (level 0) to 50°C (level 10) and were held for 3 seconds.

In the third experiment, participants were provoked with monetary subtractions (as in experiment TAPmoney) and were able to punish their opponent by administering heat stimuli (TAPmixed; as in experiment TAPheat). Parallel to experiment TAPmoney, all participants finished the experiment with 2.30€.

All experiments were implemented in the presentation software (Version 18.0, Neurobehavioral Systems, Inc., Berkeley, CA, www.neurobs.com).

### Modeling Aggressive Behavior: Mixed Effects Models

Using R (34), we fitted a linear mixed effects model on a trial-by-trial basis investigating the contribution of task-related parameters and aggressive traits on aggression for each experiment. The participant’s punishment selection in a specific trial (trial x) served as the dependent variable. The outcome of the game (win = 1 versus lose = 0) and the provocation by the opponent (0–100) in the preceding trial (x-1), as well as the AQ total score and sex (male versus female), were included as fixed effects. Continuous variables (AQ and provocation) were mean centered. The model further included random intercepts for participants to account for repeated measures. Restricted maximum likelihood was used for the estimation of variance components using the R package lme4 (35). We tested and compared two different models to ensure a good model fit. In the discarded model, we additionally included the credibility of the cover story as a random intercept (for details, see **Supplements**). To follow up significant interactions between a categorical and a continuous variable, slopes for the continuous variable were compared between the levels of the categorical variable. For interactions between two continuous variables, slopes for the first were compared while the other variable was held constant at (a) the mean − 1 standard deviation, (b) the mean, and (c) at the mean + 1 standard deviation.

Post hoc tests were calculated with the R package emmeans. Results were corrected for multiple comparisons using the Tukey method.

To compare aggressive responding across all paradigms, we conducted an exploratory analysis using z-transformed punishment selections of each modality (for details see **Supplements**).

### Emotional Reactivity

To investigate emotional reactivity to social provocation, positive and negative affect scales from the PANAS were analyzed in three separate repeated-measures Multivariate Analysis Of Variance (MANOVAs) for each experiment. Time (pre, post) served as a within-subject factor and sex (male, female) as a between-subject factor. Analyses were conducted with SPSS (IBM SPSS Statistics 25.0; Ethningen; Germany) and results were corrected for multiple comparisons using Bonferroni correction.

### Strategy

Behavior in social interactions involving repeated competitions might be guided by strategies. To examine whether participants follow a strategy and if it affects their behavior, the following analyses were performed. The percentage of participants who applied a strategy (yes versus no) was compared between experiments using the Chi Squared test. Post hoc tests were performed with adjusted residual scores as described by Beasley and Schumacher (36). To examine whether the pursuit of a strategy influenced participants punishment behavior, a univariate ANOVA with the overall punishment behavior (i.e., mean monetary reduction in experiment TAPmoney; mean intensity of heat stimuli in experiment TAPheat and TAPmixed) as the dependent variable and strategy as a fixed factor was computed separately for each experiment. Analyses were performed using SPSS and results were corrected for multiple comparisons using Bonferroni correction.

## Results

### Mixed Models

*Experiment TAPmoney:* Parameter estimates for fixed effects on the participant’s punishment selections are presented in **Table 2**. Significant effects and interactions are summarized below. Variance and standard deviation of the random intercept (participants) were 450.5 and 21.22, respectively. The estimated effect size of the model (including fixed and random effects) was R^2^ = 0.53. The linear mixed effects model showed a main effect of outcome with higher punishment selections after won trials as compared with lost trials (*t*[2192] = 2.07, *p* = .04). Results of the mixed model further indicated higher punishment selections following high provocation as compared with low (*t*(2191) = 15.555, *p* < .001). Two-way interactions between outcome and AQ (*t*(2192) = −5.563, *p* < .001), outcome and provocation (*t*(2191) = −3.213, *p* < .001), sex and provocation (*t*(2191) = −3.253, *p* < .001) and AQ and provocation (*t*(2191) = 3.258, *p* < .001) yielded significant results. Associated *post hoc* tests (**Table 3**) demonstrated that despite generally choosing higher punishments after won trials, lost competitions led to higher punishment selections, depending on trait aggression and provocation levels. Participants with high trait aggression reacted with higher punishment selections to lost reaction time competitions than individuals who scored lower on the AQ. Further, participants reacted stronger to increasing provocation following lost trials than following won trials. The interaction between provocation and AQ revealed that individuals with higher trait aggression, reacted with higher punishment selections to increasing provocation. Finally, females showed stronger reactions—in the form of high monetary punishments—to increasing provocation than males. All other predictors and interactions did not reach significance.

**Table 2:** Fixed effects of mixed model for experiment TAPmoney.

Predictor	*b*	SE	*t*	*p*
Intercept	57.35	6.17	9.300	<.001
Outcome	1.96	0.95	2.071	0.04
Provocation	0.48	0.03	15.555	<.001
AQ	0.36	0.57	0.629	0.54
Sex	13.02	8.87	1.469	0.16
Outcome × provocation	−0.11	0.03	−3.213	.001
Outcome × AQ	−0.46	0.08	−5.563	<.001
Provocation × AQ	0.01	0.00	3.258	.001
Provocation × sex	−0.11	0.03	−3.253	.001
AQ × sex	−0.62	0.78	−0.795	0.43
Outcome × provocation × AQ	0.00	0.00	1.246	0.21
Provocation × AQ × sex	−0.00	0.00	−0.630	0.53

p values calculated using Statterthwaite degrees of freedom; AQ, Aggression Questionnaire; SE, standard error.

**Table 3:** Post hoc tests of significant interactions of mixed model for experiment TAPmoney.

Significant interaction effects	Model	*b*	SE	Difference in slopes	
				*t*	*p*
Outcome × provocation	Slope for provocation when outcome = 0	0.420	0.0261		
	Slope for provocation when outcome = 1	0.314	0.0220	3.213	.001
Outcome × AQ	Slope for AQ when outcome = 0	0.0515	0.391		
	Slope for AQ when outcome = 1	−0.4060	0.389	5.563	<.001
Provocation × AQ	a) Slope for provocation when AQ = mean AQ - SD	0.256	0.0247	(a-b) −6.322	<.001
	b) Slope for provocation when AQ = mean AQ	0.367	0.0176	(a-c) −6.322	<.001
	c) Slope for provocation when AQ = mean AQ + SD	0.478	0.0249	(b-c) −6.322	<.001
Provocation × sex	Slope for provocation when sex = female	0.423	0.0241		
	Slope for provocation when sex = male	0.311	0.0252	3.253	.001

p values adjusted using the Tukey method; AQ, Aggression Questionnaire; SD, standard deviation; SE, standard error.

*Experiment TAPheat:* Parameter estimates for fixed effects are shown in **Table 4**. Significant effects and interactions are summarized below. Variance and standard deviation of the random intercept (participants) were 450.9 and 21.23, respectively. The estimated effect size of the model (including fixed and random effects) was R^2^ = 0.65. The linear mixed-effects model revealed a main effect of provocation, demonstrating higher punishment selection under high provocation than under low provocation (*t*(2016) = 11.985, *p* < .001). A significant two-way interaction was observed between sex and provocation (*t*(2016) = −2.853, *p* < .001). *Post hoc* tests indicated stronger reactions—in the form of great heat intensities—to increasing provocation in females as compared to males. Both predictors also reached significance in a three-way interaction with AQ (*t*[2016] = 2.851, *p* < .001). Associated *post hoc* comparisons (**Table 5**) demonstrated that the aforementioned interaction holds for low and average trait aggression and that sex differences were abolished in individuals with high trait aggression. Further, higher AQ was associated with greater reactivity to increasing provocation in males, whereas in females, AQ did not interact with the reaction to provocation.

**Table 4:** Fixed effects of mixed model for experiment TAPheat.

Predictor	*b*	SE	*t*	*p*
Intercept	37.37	6.71	5.569	<.001
Outcome	1.15	0.87	1.319	0.19
Provocation	0.34	0.03	11.985	<.001
AQ	0.024	0.41	0.578	0.57
Sex	13.09	9.68	1.352	0.19
Outcome × provocation	0.04	0.03	1.327	0.18
Outcome × AQ	0.00	0.05	0.089	0.93
Provocation × AQ	0.00	0.00	0.289	0.77
Provocation × sex	−0.09	0.03	−2.853	.001
AQ × gender	0.39	0.57	0.675	0.51
Outcome × provocation × AQ	0.00	0.00	0.576	0.56
Provocation × AQ × Sex	0.01	0.00	2.815	.001

p Values calculated using Statterthwaite degrees of freedom; AQ, Aggression Questionnaire; SE, standard error.

**Table 5:** Post hoc tests of significant interactions of mixed model for experiment TAPheat.

Significant interaction effects	Model	*b*	SE	Difference in slopes	
				*t* ratio	*p*
Provocation × sex	Slope for provocation when sex = female	0.360	0.0228		
	Slope for provocation when sex = male	0.266	0.0238	2.853	.004
Provocation × AQ × sex	Slope for provocation when AQ = mean AQ – SD for females	0.343	0.0258		
	Slope for provocation when AQ = mean AQ – SD for males	0.156	0.0389	4.006	<.001
	Slope for provocation when AQ = mean AQ for females	0.360	0.0228		
	Slope for provocation when AQ = mean AQ for males	0.266	0.0238	2.853	.004
	Slope for provocation when AQ = mean AQ + SD for females	0.377	0.0389		
	Slope for provocation when AQ = mean AQ + SD for males	0.376	0.0257	0.026	0.98
	Difference in slopes for provocation for mean AQ ± SD in females			−0.723	0.75
	Difference in slopes for provocation for mean AQ ± SD in males			−4.832	<.001

p values adjusted using the Tukey method; AQ, Aggression Questionnaire; SD, standard deviation; SE, standard error.

*Experiment TAPmixed:* Parameter estimates for fixed effects on the participant’s punishment selection are presented in **Table 6**. Significant effects and interactions are summarized below. Variance and standard deviation of the random intercept (participants) were 264.2 and 16.25, respectively. The estimated effect size of the model (including fixed and random effects) was R^2^ = 0.5. The linear mixed-effects model showed a main effect of outcome (*t*(2372) = −4.896, *p* < .001) with higher punishment selections following lost trials compared with won trials. Higher punishment selections were observed with increasing provocation (*t*[2368] = 9.491, *p* < .001). Males selected significantly higher punishments than females (*t*[27] = 3.647, *p* = .001). A two-way interaction was found between outcome and AQ (*t*[2369] = −2.049, *p* = .04). *Post hoc* tests indicated higher punishment selections following lost as compared with won competitions in individuals with high aggressive traits. AQ also interacted with provocation (*t*[2368] = 2.986, *p* = .001). Subsequent comparisons did not reach significance. Furthermore, a three-way interaction was found between provocation, AQ, and sex (*t*[2368] = −3.314, *p* < .001). Associated *post hoc* comparisons (**Table 7**) demonstrated stronger reactions—in the form of great heat intensities—to provocation for low trait aggression in males as compared to females. With increasing trait aggression, sex differences in the reaction to increasing provocation were abolished. Further, higher AQ was associated with greater reactivity to increasing provocation in females, whereas in males, AQ did not impact the reaction to provocation.

**Table 6:** Fixed effects of mixed model for experiment TAPmixed.

Predictor	*b*	SE	*t*	*p*
Intercept	39.97	4.38	9.118	<.001
Outcome	−4.51	0.92	−4.896	<.001
Provocation	0.28	0.03	9.491	<.001
AQ	0.23	0.30	0.770	0.45
Sex	22.33	6.12	3.647	.001
Outcome × provocation	0.01	0.03	0.229	0.82
Outcome × AQ	−0.12	0.06	−2.049	0.04
Provocation × AQ	0.01	0.00	2.986	.001
Provocation × sex	0.05	0.03	1.478	0.14
AQ × sex	0.28	0.42	0.660	0.52
Outcome × provocation × AQ	−0.00	0.00	−0.762	0.45
Provocation × AQ × sex	−0.01	0.00	−3.314	<.001

p values calculated using Statterthwaite degrees of freedom; AQ, Aggression Questionnaire; SE, standard error.

**Table 7:** Post hoc tests of significant interactions of mixed model experiment TAPmixed.

Significant interaction effects	Model	*b*	SE	Difference in slopes	
				*t* ratio	*p*
Provocation × AQ	a) Slope for provocation when AQ = mean AQ - SD	0.282	0.0224	(a-b) −1.449	0.32
	b) Slope for provocation when AQ = mean AQ	0.305	0.0159	(a-c) −1.449	0.32
	c) Slope for provocation when AQ = mean AQ + SD	0.328	0.0226	(b-c) −1.449	0.32
Outcome × AQ	Slope for AQ when outcome = 0	0.370	0.211		
	Slope for AQ when outcome = 1	0.249	0.210	2.049	0.04
Provocation × AQ × sex	Slope for provocation when AQ = mean AQ – SD for females	0.207	0.0294		
	Slope for provocation when AQ = mean AQ – SD for males	0.358	0.0336	−3.385	<.001
	Slope for provocation when AQ = mean AQ for females	0.282	0.0222		
	Slope for provocation when AQ = mean AQ for males	0.328	0.0226	−1.478	0.14
	Slope for provocation when AQ = mean AQ + SD for females	0.357	0.0340		
	Slope for provocation when AQ = mean AQ + SD for males	0.299	0.0293	1.294	0.20
	Difference in slopes for provocation for mean AQ ± SD in females			−3.298	.003
	Difference in slopes for provocation for mean AQ ± SD in males			1.328	0.38

p values adjusted using the Tukey method; AQ, Aggression Questionnaire; SD, standard deviation; SE, standard error.

**Figure 3:**
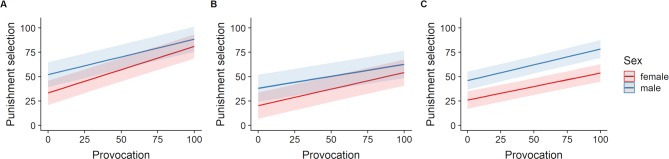
Slopes for provocation for males and females including 95% confidence intervals. **(A)** Interaction of provocation and sex in experiment TAPmoney (*p* = .001). **(B)** Interaction of provocation and sex in experiment TAPheat (*p* = .001). **(C)** Main effect of sex in experiment TAPmixed (*p* = .001).

**Figure 4:**
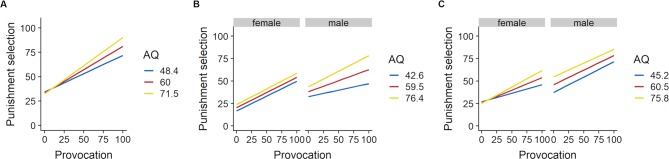
Slopes for provocation for mean AQ ± 1 SD separately for each experiment. **(A)** Interaction of provocation and AQ in experiment TAPmoney (*p* = .001). **(B)** Interaction of provocation, AQ, and sex in experiment TAPheat (*p* = .001). **(C)** Interaction of provocation, AQ and sex in experiment TAPmixed (*p* < .001). AQ, Aggression Questionnaire; SD, standard deviation.

A depiction of the association between sex and provocation and between AQ and provocation for each experiment is shown in **Figure 3** and **Figure 4**, respectively.

### Emotional Reactivity

For the TAPmoney experiment, the repeated measures MANOVA revealed a main effect of time (*F*(2, 24) = 4.816, *p* = .017), resulting in significantly increased negative feelings (*p* = .039) following the TAP. Sex had no significant influence on emotional reactivity (*F*(2, 24) = 2.3, *p* = .122). The repeated-measures MANOVA for the TAPheat experiment also showed a main effect of time (*F*(2, 24) = 11.74, *p* < .001) and a significant interaction between sex and time (*F*(2, 24) = 10.786, *p* < .001). Pairwise comparisons demonstrated that positive emotions decreased in females (*p* < .001) whereas males showed a decrease in negative emotions (*p* = .037). For the TAPmixed experiment, effects of time (*F*(2, 24) = 0.975, *p* = .392) and sex (*F*(2, 24) = 0.644, *p* = .534) did not reach significance in the repeated-measures MANOVA.

### Strategy

The percentage of participants pursuing a strategy differed between experiments (Pearson Chi-Square = 8.530, *p* = .014). *Post hoc* comparisons demonstrated that in experiment TAPheat, significantly fewer participants followed a strategy as compared with experiments TAPmoney and TAPmixed (*p* = .007).

Univariate ANOVAs showed no effect of strategy on the overall punishment behavior in experiment TAPmoney (*F*(1, 25) = 0.124, *p* = .728), experiment TAPheat (*F*(1, 24) = 0.064, *p* = .802), and experiment TAPmixed (*F*(1, 25) = 0.072, *p* = .790).

## Discussion

In the current study, we investigated the effects of contextual and personality factors on aggressive responding in a well-known standardized experimental aggression paradigm, the TAP. To examine these influences across different provocation and punishment modalities, three experiments used different pairings of non-physical and physical provocation and punishment stimuli. Overall, we could provide evidence for provocation-induced aggression in all three experiments, which is likely to be enhanced by trait aggression. Another contextual factor, i.e., losing against the opponent, seems only important in competitions for money. In these situations, losing the competition leads high aggressive individuals to select higher punishments. All three experiments indicate that women behave less aggressively than men; however, under high provocation, these differences decrease. In situations including physical aggression, sex differences in reactivity to provocation further depend on trait aggression and disappear for highly aggressive individuals.

### Provocation

The current results underline the modificatory power of contextual and personality factors on aggressive reactions as specified in the GAM. Among many possible contributing factors, including biological variability, one of the most relevant factors is provocation. In this study, we could show that different variants of provocation effectively increased aggressive responding. Throughout all modalities, increasing provocation led participants to respond more aggressively as observed in higher monetary reductions or higher intensities of heat stimulation. This behavior, described as “tit-for-tat” (18) seems to be a universal reaction, which can be observed for different provocation or punishment modalities and even persists if modalities do not match. The “tit-for tat” strategy has been frequently observed in previous studies ([e.g., Refs. (10–12, 18, 37).

Despite its seemingly universal nature, the reaction to provocation is influenced by personality traits, as well as other contextual factors. In the TAPmoney and TAPmixed version, we could demonstrate that reactivity to provocation in non-physical aggression settings (monetary subtractions) was further enhanced by losing the prior reaction time task. Additionally, aggressive traits impacted the strength of reaction to provocation. It is further noteworthy that the overall level of punishment selections appears to be higher in non-physical scenarios (TAPmoney) as compared with settings involving physical aggression (TAPheat and TAPmixed). This might indicate that even though the reaction to provocation is evident when money subtractions and when heat stimulation is used as provocation, the threshold for initial aggressive behavior is higher when individuals can apply physical punishments such as heat.

### Sex Differences

Next to provocation, participants’ sex seems to be another universal influence as this was observed in all three experiments. Overall, results demonstrate higher levels of aggression in men as compared with women. However, in experiments TAPmoney and TAPheat, in which provocation and punishment modality were identical, women reacted stronger to the increase in provocation than men. In aggression research, it has been frequently illustrated that men and women differ with respect to their aggressive behavior. Meta-analyses not only reported higher aggression in males compared with females (19, 20, 38) but also that sex differences dissolve under provocation (19), and women gradually increase punishment selections (38). This led to the assumption that women might become aggressive after prolonged provocation.

Substantiating these earlier research findings, women’s aggressive reactions approximated those of men’s following high provocation. Notably, we used a continuous increase in provocation adapted from a previous study (37). Hence, although the strong increase in punishment selections in females is highly linked to high levels of preceding provocation, a prolonged exposure could also contribute to the decrease in sex differences toward the end of the paradigm. Due to increasing punishment levels over time, the current version of the paradigm might not allow a final conclusion regarding the background for the observed sex differences. It might be due to prolonged exposure or due to higher provocation levels. Although we cannot differentiate between time effects and provocation intensity effects here, provocation seems to gradually compensate the initial difference observed in males and females. We here propose that this could result from a higher threshold in women to engage in aggressive behavior. Nevertheless, future studies are needed to target the particular question of whether women react stronger to high levels of provocation or to prolonged exposure.

Importantly, in contrast to our hypotheses and to what has been reported before, sex differences were observed throughout different provocation contexts. Previous research on sex differences suggested that differences are larger for physical aggression (19, 20) and are eliminated for indirect aggression (21, 22). Here, we investigated physical and non-physical forms and demonstrated that under high provocation, sex differences are diminished as long as provocation and punishment modality are identical. A recent study investigated the neural underpinnings of sex differences, also using monetary provocation and punishment in the TAP. The study reported no differences in aggressive behavior between men and women (39). This apparent inconsistency might be attributable to the difference in the data analyses. By using a trial-by-trial approach, the current analysis is sensitive to the detection of sex differences in reactivity to provocation across all trials and provocation levels.

Persistent sex differences only existed in the TAPmixed experiment, in which monetary subtractions were used as provocation and heat stimuli as punishment. This could indicate that women might be more tentative to punish their opponent following a “tit-for-tat” strategy in cases of unequal and/or more severe forms of aggression (i.e., physical pain).

### Trait Aggression

As suggested by the GAM, personality traits have a high impact on aggressive behavior and consequently, trait aggression is among the most frequently studied predictors of aggressive behavior. Naturally, and as shown in all the three experiments of this study, highly aggressive individuals respond more aggressively than individuals characterized by low aggressive traits (23, 40). Individuals sensitive to provocation not only react stronger to provocation but also show higher overt aggression (41). Possibly, these individuals are primarily characterized by lower self-control.

Results of the TAPheat and TAPmixed version revealed that when physical aggression is involved, the impact of trait aggressiveness on reactivity to provocation differs between men and women. In the TAPmixed version, in which non-physical provocation and physical punishment are paired, trait aggression further boosts the aggressive response to provocation in women, such that with increasing aggressiveness, they show similar reactivity to provocation as men. It, thus, appears that trait aggression lowers the threshold for aggressive responses in females, who generally appear to be more tentative to physically punish an opponent who did not provoke them in the same way.

### Outcome

In addition to provocation, we investigated the predictive capabilities of the outcome of the reaction time competitions for aggressive behavior. Although analyses in the TAPmoney version revealed higher monetary subtractions following won trials as compared with lost trials, which might be explained by a simultaneous increase of won competitions and increase in provocation intensity with time, in the following, we will focus on the discussion of reported interactions.

Losing can be a strongly frustrating event, especially if it occurs repeatedly and has the potential to trigger norm violations (42). Interestingly, the outcome of the repeated reaction time game contributed only to aggression when money was involved in the social interaction. We found that in experiments TAPmoney and TAPmixed, individuals with high trait aggressiveness reacted stronger to lost competitions than participants with low aggressive traits. Furthermore, in the TAPmoney version, losing the prior reaction time competition increased aggressive responding to provocation. Previously, it has been reported that the punishment selection (noise blasts) was not affected by the outcome of the prior reaction time game (43). Consistently, we found no effect of the outcome using physical provocation and punishment, thus suggesting that outcome primarily affects behaviors that do not involve physical aggression. Therefore, results might indicate that in competitions for money, losing implies profit for the opponent and loss of money for oneself. Hence, losing the reaction time game could present an additional frustrating component, thus, causing participants to react stronger to provocation after losing the competition.

We further investigated strategic aims of participants in the social provocation task. Interestingly, even though across all experiments, pursuing a strategy did not directly influence aggressive responding, participants more frequently applied a strategy when monetary gain/loss were involved. These results suggest that participants are more likely to follow strategies when competing against an opponent for money and less likely, when only physical provocation and punishment are involved.

### Emotional Reactivity

Overall, results of the TAPmoney and TAPheat versions indicate that engaging in a social provocation task negatively affects the emotional state. These shifts in emotions might differ among men and women as indicated by the current study. Importantly, interpretation of these rather unspecific scales (positive and negative affect) is limited. It is possible that in social interactions including painful punishment, emotions associated with anxiety or fear play a larger role as compared with situations with monetary punishments. Emotional reactions toward provocation may be specifically useful to study when investigating, for instance, interventions, such as emotion regulation and underlying cognitive or biological mechanisms [e.g., Refs. (39, 44)]. Studies might profit from a more specific approach to study particular emotions repeatedly during aggressive interactions.

In experiment TAPmixed, no changes in emotions were found. It is noteworthy that effect sizes might be too small to detect significant changes in small samples as investigated here. Specifically for the investigation of sex differences, future studies might profit from larger sample sizes.

### Comments on Different Versions

Within the PSAP, common implementations use money as a provoking and punishment stimulus, whereas most versions of the TAP apply physical stimulus, such as an aversive noise. Results of the study at hand suggest that individuals may generally display a lower threshold for aggression if the punishment modality is money as compared to the infliction of physical harm. The provocation effect, however, can be observed across all experimental versions investigated in this study. Furthermore, implementing the paradigm using money provides a substantial advantage within settings using magnetic resonance imaging (MRI). It is simpler to implement cash withdrawal than to inflict physical pain in such settings. Especially with the frequently used noise blast, difficulties with the loud noises emerging from the MRI might occur. From a conceptual point of view, physical provocation and punishment are more closely linked to aggression occurring outside the laboratory. In the past, it has often been criticized that aggression measured with the TAP using electric shocks lacks construct validity and does not entirely relate to aggression displayed in real-life settings (45). However, the current results underline the effectivity of the task, and, moreover provide some evidence for general effects independent of the provocation and punishment modality.

### Limitations

For the investigation of sex effects, the sample size of each experiment might be too small to detect weak effects. Larger studies are needed to replicate and extend the current findings. Moreover, the current version of the TAP does not allow a differentiation between provocation intensity and exposure. Future studies might explore whether sex differences in the reaction to provocation, as seen in this study, rather depend on the intensity of the provocation or the length of exposure.

### Conclusion

The present study contributes to the investigation of aggressive behavior across different punishment modalities and further examines the influence of non-physical provocation on the infliction of physical punishment. We show that physical and non-physical provocations are strong predictors of aggressive responding and further that this reactivity is strengthened by high trait aggressiveness. Women seem to have a higher threshold to react to provocation but this might be passed by prolonged, increasing provocation. In contexts involving physical aggression, these sex differences are moderated by aggressive traits and partly eliminated. Loss, or the frustration of losing a competition, seems to be an effective trigger for aggressive responding when competing for money. Overall, the current study substantiates existing evidence and provides information about the contribution of trait aggression, sex, provocation, and the outcome of competitions to aggressive behavior across situations, including physical and non-physical aggression.

## Data Availability

The datasets for this study will not be made publicly available because we do not have an ethics votum for sharing the data.

## Ethics Statement

All subjects gave written informed consent in accordance with the Declaration of Helsinki. The protocol was approved by the internal review board of the medical faculty of the RWTH Aachen.

## Author Contributions

CW, UH, FS, JB, and LW contributed conception and design of the study. CW, DA, and LW acquired the data. CW, PH, and LW were involved in the statistical analysis. CW, UH, PH, and LW contributed to the data interpretation. CW wrote the first draft of the manuscript. All authors read and approved the submitted version.

## Funding

This study was supported by the German Research Foundation (DFG, IRTG2150).

## Conflict of Interest Statement

The authors declare that the research was conducted in the absence of any commercial or financial relationships that could be construed as a potential conflict of interest.
