# Supplementary material for: On the Complexity of Aggressive Behavior: Contextual and Individual Factors in the Taylor Aggression Paradigm
Source: Front Psychiatry. 2019 Jul 25;10:521. doi: 10.3389/fpsyt.2019.00521 (PMC6669806; doi:10.3389/fpsyt.2019.00521)
Supplement: Supplementary file 1 [file DataSheet_1.docx]

Supplementary Material

# Supplementary Data

*Model selection*

Comparison of two linear mixed effects models was performed using a Chi Square test for each experiment. Results revealed that including the belief in the cover story as a random intercept did not show a significant difference in reduction in the sum of squares in experiment TAPmoney (*p* = .99), experiment TAPheat (*p* = .99) and experiment TAPmixed (*p* = .99). Based on these results, the simpler model (without belief as a random intercept) was used for subsequent analyses.

*Comparison experiments*

To compare punishment selections across experiments, mean punishment selections were z-transformed and subsequently compared using a univariate analysis of variance (ANOVA) with the between-subject factor experiment (TAPmoney, TAPheat, TAPmixed). The ANOVA showed that punishment selections differed significantly between experiments (*F*(2,78) = 5.114, *p* = .005). Associated post hoc comparisons demonstrated that in experiment TAPmoney participants chose higher punishments than in experiment TAPheat (*p* = .007) and experiment TAPmixed (*p* = .033). All other comparisons did not reach significance. Pairwise comparisons were corrected for multiple comparison using Bonferroni correction.
